# Functional rejuvenation of endothelial cell aging by transient reprogramming

**DOI:** 10.1007/s00395-026-01192-7

**Published:** 2026-06-26

**Authors:** Katrin Kalies, Kai Knoepp, Laura Hehl, Simon Guerlach, Laura Sandiano, Andreas Simm, Jochen Dutzmann, Daniel Sedding

**Affiliations:** 1 Mid-German Heart Center, Department of Internal Medicine III, Division of Cardiology, Angiology and Intensive Medical Care, University Hospital Halle, Martin-Luther-University Halle-Wittenberg, Ernst-Grube-Strasse 40, 06120 Halle (Saale), Germany; 2 Department of Cardiology and Angiology, Hannover Medical School, Carl-Neuberg Straße 1, 30625 Hannover, Germany; 3 Department for Cardiac Surgery, University Hospital Halle, Martin-Luther-University Halle-Wittenberg, Ernst-Grube-Strasse 40, 06120 Halle (Saale), Germany

**Keywords:** Endothelial dysfunction, Senescence, Cellular reprogramming, Angiogenesis

## Abstract

**Abstract:**

Senescent endothelial cells (ECs), characterized by a reduced angiogenic and regenerative potential, are key players in the pathophysiology of cardiovascular diseases. Therefore, targeting these cells has been suggested as an effective therapeutic strategy to increase health. Here, we are the first to report that non-genetic overexpression of the Yamanaka factors induces partial functional rejuvenation and attenuation of senescence-associated features in endothelial cells. Methods to characterize the effects of the transient reprogramming included quantification of gene expression as well as measurements of cellular functions *in vitro*. Further, *in vivo* experiments were performed in a hind-limb ischemia model. The application of the pharmacological cocktail to replicative senescent ECs resulted in a robust but timely restricted activation of Oct3/4, Sox2, Klf4, and c-Myc (*p* < 0.0 and *p* < 0.01). This was associated with a significant reduction of senescence markers such as p16ink4a and p14arf (*p* < 0.01). Additionally, qPCR-based telomere length measurements were stabilized, and functional properties of senescent ECs, such as proliferation, migration, sprouting, and tube formation, were improved (*p* < 0.05). Continuous cultivation of the treated cells over the long term indicated that expression of p16ink4a and p14arf remained significantly low, while cell migration remained enhanced. *In vivo*, a significantly improved blood flow was observed at 7 and 14 days after hind-limb ischemia in 21 months old C57BL/6 mice (*p* < 0.001). In conclusion, we revealed that a partial attenuation of endothelial cell senescence-associated features can be induced by a short pharmacological overexpression of the Yamanaka factors. While the compounds used are individually approved for other indications, their combined use in this context highlights a conceptual translational potential, whereas the clinical applicability of this approach remains to be evaluated.

**Graphical abstract:**

**Figure:**
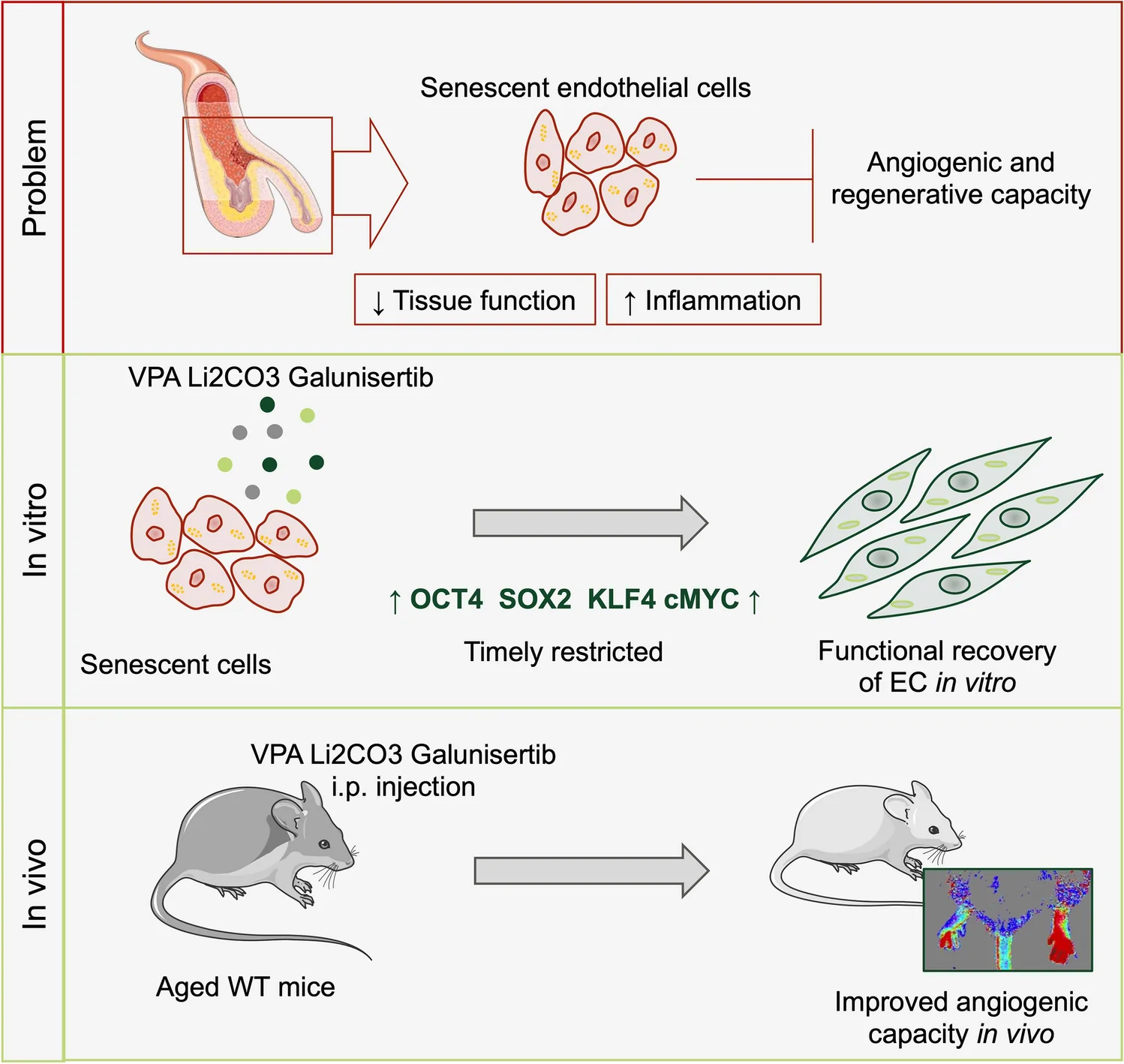

**Supplementary Information:**

The online version contains supplementary material available at https://doi.org/10.1007/s00395-026-01192-7.

## Introduction

Cardiovascular diseases (CVD), such as coronary heart disease and heart failure, are characterized by vascular impairment and are the leading cause of death worldwide [20, 60]. This vascular impairment is clinically characterized by the increased size and stiffness of the large arteries, elevated blood pressure, or loss of vascular density [20]. Among other CVD risk factors such as hypertension, dyslipidemia, and chronic noxa, age is the most important independent risk factor [16, 46]. So far, essential progress has been made in the treatment of CVD, but mortality remains at a high level, which means that the clinical need for therapeutic interventions remains. Additionally, there is a strong relation to the increased life expectancy contributing to still high incidence of CVD [16]. Besides impaired microvascular perfusion, inflammation, and oxidative stress, a major cause of vascular impairment is the development of endothelial dysfunction leading to a loss of regenerative and angiogenic capacity [9, 20, 22, 31, 49, 58]. Consistent with this concept, endothelial cell-specific regulatory mechanisms have been shown to critically govern angiogenic function and vascular adaptation. Against this background, strategies aimed at restoring endothelial function and angiogenic capacity are of particular interest, especially in the setting of age-associated vascular changes [8, 31]. A causal explanation for these age-associated changes is the accumulation of senescent cells over the life span in healthy arterial tissue [36, 39], as well as in atherosclerotic plaques of ischemic heart disease [38] and heart failure [24]. In general, cellular senescence is described as cellular aging, gradually leading to a permanent cell cycle arrest in the G0/G1-phase [13, 17, 19] most often initiated by the p53 or p16/pRB pathway [12, 21]. So far, target options include senolytic treatment [4], enhancing the immune response [28, 33], increasing the replicative potential [2, 10], and cellular reprogramming by viral vectors [41]. Recent studies show that viral overexpression of OCT3/4, SOX2, KLF4, and cMYC (OSKM) can also reverse the age-associated phenotype, but this approach also carries the risk of genomic instability [35]. Intriguingly, studies in different non-senescent cell types indicate that cellular reprogramming can also be achieved and supported by a mixture of chemical compounds with the advantage of a safer application in a time- and concentration-dependent manner [61].

In the present study, we demonstrate for the first time that a pharmacological cocktail composed of compounds individually approved by the FDA for other indications can induce a temporally restricted overexpression of the Yamanaka factors (OSKM), resulting in partial functional rejuvenation of senescent endothelial cells and improved endothelial function and angiogenic capacity *in vitro* and *in vivo*. These three substances include valproic acid, a fatty acid derivate, and histone deacetylase-1 inhibitor [3, 32, 45], lithium carbonate, having mainly glycogen synthase kinase 3 as a target [14, 47, 50], and galunisertib, an antineoplastic antagonist of the tyrosine kinase transforming growth factor-beta receptor type 1 [25].

## Materials and methods

Further information can be found in the supplementary material.

### Cell culture

All *in vitro* experiments were performed on human umbilical vein endothelial cells purchased from Lonza. Cells were cultivated in an endothelial growth medium (ECGM 2, PromoCell) at 37 °C with 5% CO_2_ in a humidified incubator, grown to confluency, and passaged in a 1:3 ratio. Cells at passage <5 were claimed as non-senescent, and cells at passage > 14 were claimed as replicative senescent cells. Cells were characterized for replicative senescence before experiments as previously described [27].

### Induction of cellular damage and pharmacological reprogramming

Cellular damage was induced by treatment with 50 µM hydrogen peroxide for 24 h. For the pharmacological reprogramming, cells were treated with 0.3 mM Li2CO3 (Sigma), 0.5 mM valproic acid (Sigma), and 1 µM Galunisertib (Selleckchem). Treatment was performed for 72 h, then the media were changed, and consecutive analyses were performed. For long-term assays, cells were further cultivated for 28 days after the initial treatment, with frequent medium changes but without refreshing the pharmacological treatment.

### siRNA transfection

Transfection of the cells with siRNA was performed using RNAiMax (Thermo Fisher Scientific) and Opti-MEM (Thermo Fisher Scientific) according to the manufacturer’s protocol. Briefly, siRNA and scrambled controls (see Suppl Table S1) were mixed with Lipofectamine RNAiMax and shortly incubated in Opti-MEM media. Cells were seeded in antibiotic-free media and treated with the liposomal complexes. The final concentration of siRNA was 20 nM. The efficiency of siRNA knockdown was verified by qRT-PCR. 

### RNA isolation, reverse transcription, and qRT-PCR

Total RNA was isolated using the Plus RNeasy Mini Kit (Qiagen) according to the manufacturer’s protocol. Briefly, cells were washed with PBS (3x), lysed, genomic DNA was removed, and RNA was eluted through a column-based isolation. RNA was transcribed to cDNA using the High-Capacity cDNA Reverse Transcription Kit (Thermo Fisher Scientific) and qRT-PCR analysis was performed with the Blue S’Green qPCR Kit (Biozym). Both kits were used according to the manufacturer’s instructions with an input of 100 ng total RNA. A list of used primers is shown in Supplementary Table S2.

### Protein isolation and Western blot analysis

Proteins were isolated with RIPA buffer supplemented with 10% protease-inhibitor cocktail and 1% PMSF on ice (20 min) and then centrifuged. Protein concentration was determined with a Bradford assay. For gel electrophoresis, 30 ng protein was loaded onto precast gradient gels (Biorad). Proteins were transferred onto a PVDF membrane with a tank-blot system. A list of used antibodies can be found in Supplemental Table S3.

### DNA isolation and determination of telomere length

Genomic DNA was isolated following the manufacturer’s instructions with the GeneJET Genomic DNA Purification Kit (Thermo Fisher Scientific) which works on a column-based isolation technique including a treatment with Proteinase K and RNase A. Telomere length was determined via qRT-PCR analysis based on the Relative Human Telomere Length Quantification assay (ScienCell Research Laboratories).

### Immunofluorescence staining of cells *in vitro*

For immunofluorescence staining of the cells, the cells were seeded in eight-well chamber slides. After adherence, cells were fixed for 30 min, blocked, and incubated with the primary antibody overnight. On day 2, slides were washed and incubated with the secondary antibody for 2 h. After additional washing steps, the slides were mounted with DAPI-containing mounting medium, closed with a cover slip, and sealed. A list of used antibodies can be found in the Supplemental Table S3.

### Isolation of endothelial cells from mice samples

An endothelial-enriched cell fraction was isolated from murine samples via CD146 + magnetic beads. The tissue was cut into small pieces, enzymatically digested, and homogenized using the Multi Tissue Dissociation Kit and gentleMACS Dissociator as per the manufacturer’s guidelines. The tissue fragments were placed in an enzyme mixture within a C tube and homogenized for 1 h at 37 °C. The resulting cell suspension was filtered through a 70 μm strainer, and debris was removed using gradient centrifugation with the Debris Removal Kit. The cell suspension was mixed with cold PBS, layered with the debris removal solution, gently mixed, and then overlaid with PBS. The mixture was centrifuged at 3000xg for 10 min, after which the top two phases were removed, and the bottom phase was washed with PBS. Erythrocytes were lysed using the Red Blood Cell Lysis Kit; the cell pellet was incubated with a lysis solution for 2 min, briefly centrifuged, and the reaction was terminated with an enzyme mix. Finally, the cells were incubated with CD146 magnetic beads for 15 min, separated using MS columns and a MACS separator, and eluted from the column by pushing the plunger. The cell suspension was centrifuged, and the pellet was resuspended in Trizol for total RNA extraction.

### Proliferation, migration, and assessment of mitochondrial superoxide

Proliferation was assessed by cell counting using a live-cell imaging approach. Cells were seeded to subconfluency and images were taken every 30 min with a live-cell imaging system over a time period of 24 h. The number of cells was counted in every brightfield image. Additionally, proliferation was quantified by a BrdU incorporation assay (Roche). Cells were seeded in growth medium in 96-well plates to subconfluency. After the adhesion of the cells, the medium was changed to basal medium. On the second day, the cells were labeled with 10 μM of the BrdU labeling solution and incubated for 24 h. The medium was aspirated, the cells were fixed and incubated with BrdU-working solution for 90 min. After several washing steps, the substrate solution was added to the wells and incubated at room temperature until the blue color developed sufficiently for photometric detection. The reaction was stopped with a stopping solution, and absorbance was measured at 450 nm.

Migration capacity was determined by a scratch-wound assay. Cells were seeded to confluency, and a scratch wound was done with a pipette tip. Images were taken every 30 min over 24 h with a live-cell imaging system. Scratch area and cell area were determined for every image.

The amount of mitochondrial superoxide was determined with the MitoSOX Red fluorescent dye (Thermo Fisher Scientific). Cells were seeded to subconfluency and treated with the MitoSOX Red fluorescent dye according to the manufacturer’s instructions. For validation of mitochondrial superoxide specificity, cells were treated with rotenone (1 µM for 30 min) or MitoTEMPO (10 µM for 60 min) as positive and negative controls for MitoSOX fluorescence (Supplemental Fig. 1). Brightfield and red fluorescent images were taken, and red fluorescent cells were counted in relation to cells in the brightfield channel.

All experiments were performed with the live-cell imaging system Cytation 1 (Biotek) and image analysis was performed with the software Gen 5 (Biotek).

### *In vitro* angiogenesis assays

For the sprouting assay, cells were seeded with a density of 4*10^5 cells/ml in 80% growth-medium and 20% methylcellulose into hanging droplets for 24 h to allow sphere formation. On day 2, a bottom matrix was pre-prepared (50% of 2 mg/ml collagen, 10% M199 Medium, and 40% methylcellulose-stock solution) and polymerization was allowed for 1 h at 37 °C. For the top matrix, spheroids were collected, centrifuged, and resuspended in FCS and further mixed with 40% methylcellulose and 50% collagen. Polymerization was allowed for 2 h, then the matrix was overlaid with medium. Formation of sprouts was controlled by microscopy.

The tube formation assay was performed using the “In vitro Angiogenesis Assay Kit” (Cultrex) according to the manufacturer’s protocol. Briefly, plates were coated with a basement membrane followed by a polymerization step. Up to 10.000 cells were seeded into one well of the plate, and the plate was further incubated in the live-cell imaging system Cytation 1 to observe the formation of tubular networks. Images were taken every hour for 18 h. The final image was used to assess the tubular network. The tubular network was analyzed with the Angiogenesis Analyzer for ImageJ developed by G. Carpentier, M. Martinelli M, J. Courty and I. Cascone (2012) and is available online at http://image.bio.methods.free.fr/ImageJ/?Angiogenesis-Analyzer-for-ImageJ&artpage=6-6&lang=en providing more information.

### Animal experiments

All animal experiments were approved by the local authorities (Landesverwaltungsamt Sachsen-Anhalt MLU 42502–2-1623). They were performed according to the guidelines of the Federation for Laboratory Animal Science Associations and complied with the Directive 2010/63/EU of the European Parliament.

All *in vivo* experiments were performed on adult C57BL/6 mice of different ages from Janvier.

### Murine hind-limb ischemia model

To evaluate the efficiency of the pharmacological reprogramming cocktail on angiogenesis, a surgical ligation of the femoral artery was performed in two aged groups of C57BL/6 J mice (3 months or 21 months). Mice were anesthetized by inhalation of isoflurane (Forane, inhalation anesthesia, 0.25–2.0 vol% with O2 flow of 0.2 l/min). The femoral artery was ligated directly distally of the deep branch of the A. profunda femoris leading to ischemia in the gastrocnemius muscle. Laser-Doppler images were taken directly after the intervention and on day 1, 3, 5, 7 and 14 of the ligated as well as of the not ligated limb. Additionally, the mice received an injection of the pharmacological reprogramming cocktail directly after surgical intervention, as well as on days 1 and 3. Mice were injected with 100 mg/kg VPA, 60 mg/kg Li2CO3, and 30 mg/kg Galunisertib via intraperitoneal injection. At day 14, mice were euthanized by cervical dislocation/inhalation of carbon dioxide in accordance with the AVMA guidelines for the euthanasia of animals. After severing the inferior *vena cava*, the mice were perfused with PBS via the left ventricle. Organs as well as the gastrocnemius and semimembranosus muscles were collected for either tissue sections or RNA analysis.

### Immunofluorescence staining of the gastrocnemius muscle for capillary density

Immunofluorescence stainings were performed on tissue sections of 6 µm depth. Slides were fixed for 30 min, blocked, and incubated with the primary antibody for CD31 (Cell Signaling, 3528S, 1:100) overnight. On day 2, slides were washed and incubated with the secondary antibody for 2 h (Thermo Fisher Scientific, Alexa Fluor 488, 1:200). After additional washing steps, the slides were mounted with DAPI-containing mounting medium, closed with a cover slip, and sealed. Capillary density was assessed by counting CD31-positive cells relative to the total DAPI count.

### Statistical analysis

Datasets were analyzed using GraphPad Prism 8. All data are represented as mean ± standard deviation. Unless stated otherwise, n refers to the number of biological replicates derived from independent experiments. Within each biological replicate, multiple technical measurements were performed as appropriate and averaged before statistical analysis. All datasets were normalized to the respective control group and tested for variance. Statistical significance was assessed using unpaired Student’s *t *test, and one-way ANOVA, as indicated. Statistical significance is displayed in the figures using asterisks corresponding to defined *p* value thresholds.

## Results

### Cellular damages induce regeneration genes exclusively in non-senescent endothelial cells

**Fig. 1:**
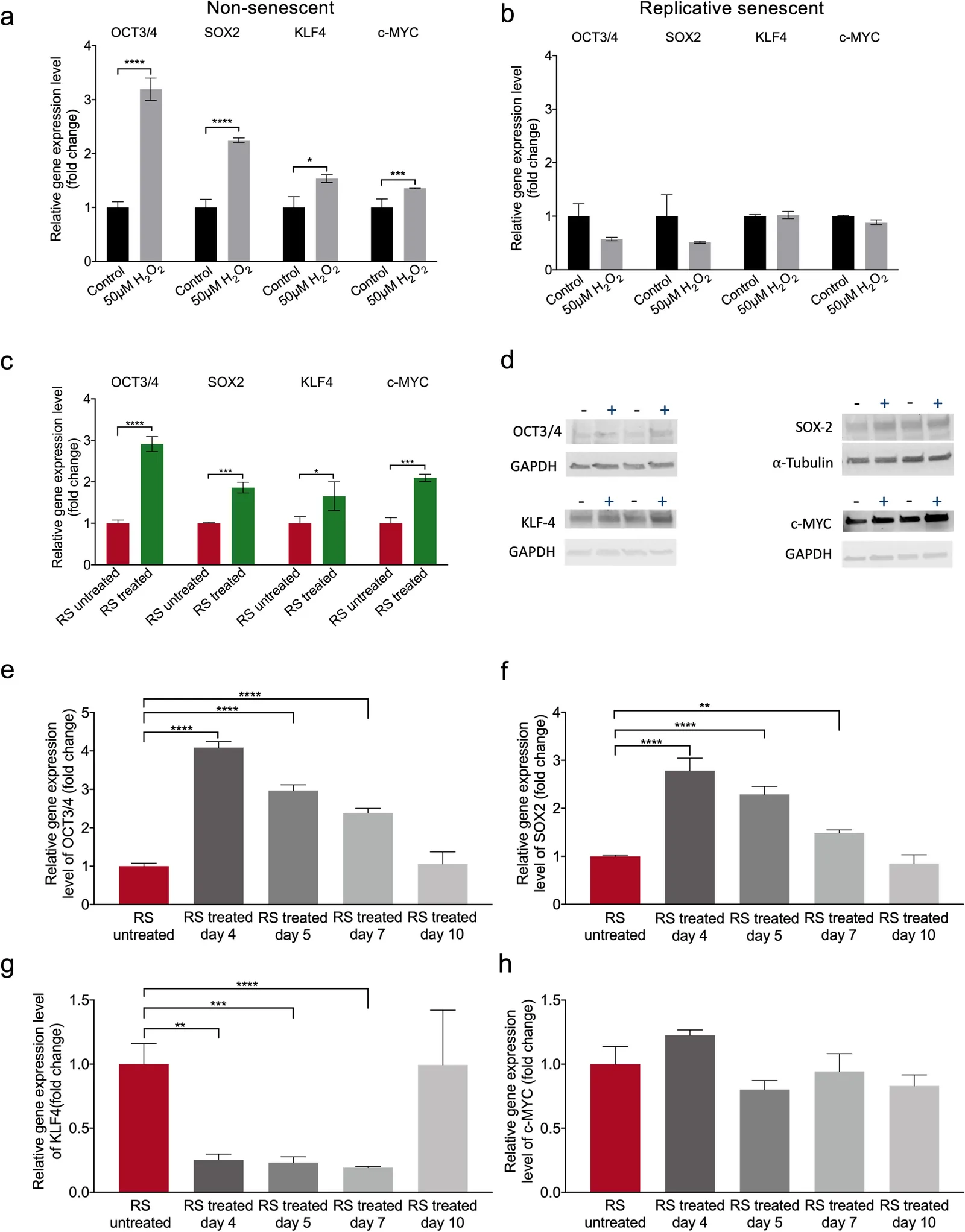
Quantification of OSKM expression after induction of endothelial cell damage with 50 μM H2O2 in non-senescent and replicative senescent EC. **a** Analysis of mRNA expression levels shows significant upregulation of OSKM in the non-senescent cells whereas **(b)** in the replicative senescent cells, no regulation is observable (n = 3 biological replicates; Student’s *t *test). The pharmacological treatment with VPA, Li2CO3, and Galunisertib leads to an application-dependent, timely restricted overexpression of OSKM in replicative senescent (RS) endothelial cells as shown in **(c)** on mRNA expression levels 72 h after initial treatment (n = 3 biological replicates; Student’s *t *test). **d** Western blot analysis confirms the overexpression of OSKM also on protein level (+ treated,—untreated) **(e–h)** Quantification of OSKM mRNA expression levels at days 4, 5, 7, and 10 after initial treatment when treatment is removed on day 3, confirms that OSKM expression slowly drops down after removal of the cocktail (*n* = 3 biological replicates; Student’s *t *test to control RS untreated). Statistical significance is indicated in the figures (*p < 0.05, **p < 0.01, ***p < 0.001, ****p < 0.0001)

The reprogramming strategy described here is based on the theory that senescent cells cannot regenerate themselves. The so-called Yamanaka- factors OCT3/4, SOX2, KLF4, and c-MYC (OSKM) are the main factors for this regeneration process. To see whether ECs and particularly senescent ECs possess regenerative capacity after cellular damage, we first investigated OSKM factor expression in ECs in a cellular damage model mimicking pathophysiological situations using hydrogen peroxide. As expected, we could observe that non-senescent ECs react with a significant upregulation of OCT3/4, SOX2, c-MYC, and KLF4. In contrast, the replicative senescent cells showed no change in the expression of OSKM (Fig. 1a and b). Taken together, we could demonstrate that non-senescent cells initiate a reprogramming process after cellular damage, whereas the senescent cells do not show OSKM expression. A therapeutic strategy might now intervene at this point in senescent cells and support the physiological impulse observable in non-senescent cells.

### Establishment of a pharmacological reprogramming strategy to induce cellular regeneration

One requirement for a feasible reprogramming strategy of replicative senescent cells is that the overexpression of the Yamanaka factors is applicable on a time-restricted basis, presenting no risk of genomic integration or initiation of teratoma formation. Several small compounds capable of promoting OSKM-associated cellular reprogramming have been approved for other clinical indications and are well characterized for their pharmacokinetics, pharmacodynamics, and tolerability (64). From these, a pharmacological cocktail of three chemicals, namely valproic acid (VPA), lithium carbonate (Li_2_CO_3_), and galunisertib (Gal), was tested for efficiency in inducing OSKM expression in replicative senescent endothelial cells. We could show that a 72-h application period to senescent ECs resulted in a robust expression of OCT3/4, SOX2, KLF-4, and c-MYC at mRNA and protein levels (Fig. 1c and d). Subsequent removal of the cocktail led to a time-dependent decline in OSKM expression. Whereas expression of c-MYC dropped directly after the removal of the pharmacological compounds, the expression of OCT3/4 and SOX2 was reduced back to baseline on day 10 after treatment (Fig. 1e–h). Interestingly, KLF-4 was initially downregulated upon removal of the pharmacological cocktail but reached a comparable expression level to that of untreated cells on day 10. Based on these results, all subsequent assays to assess improvements in cellular function were performed on day 10 after treatment. Taken together, we could thereby show that one initial treatment is sufficient to induce overexpression of OSKM in replication-induced senescent ECs, but as soon as the cocktail is removed, expression levels drop at factor-specific rates and reach a physiological level at the latest by day 10. Observation of cell morphology showed no influence of the treatment on the cells (Supplemental Fig. 2a). Staining for the endothelial cell marker CD31 confirmed that throughout the application of the pharmacological reprogramming cocktail, the cells do not lose their endothelial cell identity (Supplemental Fig. 2b and c). Further, quantification of nuclear size showed no differences (Supplemental Fig. 2d).

### The pharmacological reprogramming cocktail improves proliferation, influences the cell cycle arrest machinery, and stabilizes telomere length *in vitro* in senescent ECs

**Fig. 2:**
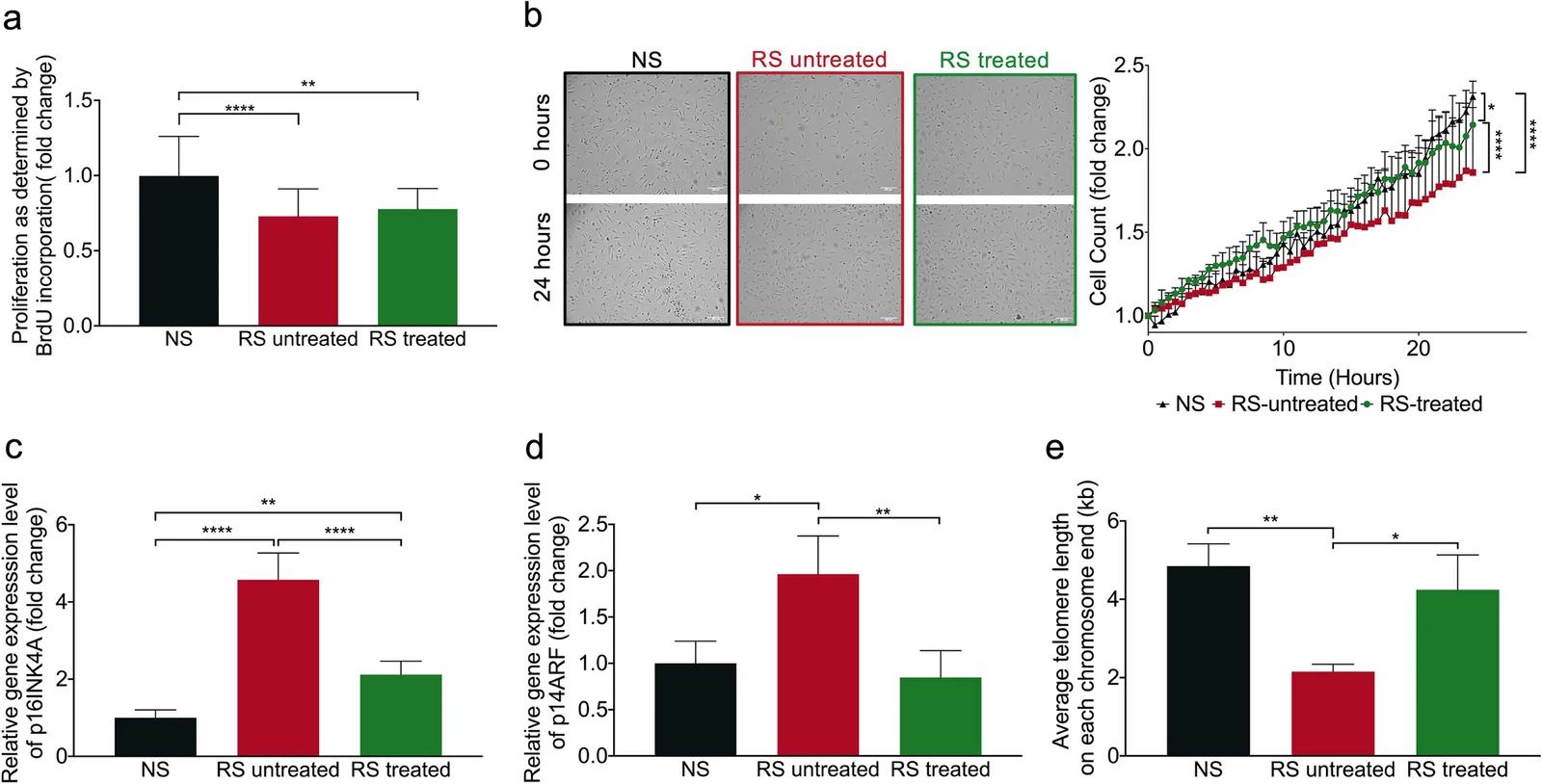
A treatment of replicative senescent endothelial cells with the pharmacological reprogramming cocktail (RS treated) improves proliferation, expression of cell cycle regulators p16INK4a and p14ARF and stabilizes telomere length in comparison to replicative senescent untreated (RS untreated) endothelial cells. **a** and **b** Determination ***of proliferation by a BrdU incorporation assay and a live-cell imaging approach. Even though BrdU incorporation does not show a significant improvement, actual cell count analysis shows the improvement of proliferation by the treatment (scale bar 200 µm. *n* = 3 biological replicates; one-way ANOVA). **c** mRNA expression analysis of p16INK4A shows significantly reduced levels in the treated cells compared to the untreated cells (*n* = 4 biological replicates; one-way ANOVA). **d** The relative gene expression level of p14ARF can be significantly reduced by the pharmacological treatment leading to an expression level comparable to non-senescent endothelial cells (*n* = 4 biological replicates; one-way ANOVA). **e** The average telomere length on each chromosome end can be stabilized and significantly extended by the pharmacological treatment (*n* = 3 biological replicates; one-way ANOVA). Statistical significance is indicated in the figures (*p < 0.05, **p < 0.01, ***p < 0.001, ****p < 0.0001)

Next, we tested the above-mentioned protocol for actual reprogramming effects on EC proliferation and the cell cycle arrest machinery *in vitro*. All assays were performed on pharmacologically treated replicative senescent cells in comparison to untreated senescent cells and a non-senescent control. Proliferation was measured based on two techniques: BrdU incorporation and cell count in a live-cell imaging approach. As expected, both techniques verified a significant impairment of proliferation in the replicative senescent cells compared to the non-senescent cells (Fig. 2a and b). Application of the cocktail, however, improved the proliferation of the senescent-treated cells, as quantified with the live-cell count assay. A similar trend was observed using the BrdU assay, though not significant. Intriguingly, cells still have a better proliferation effect when treated once and cultivated for a longer period of 28 days (Supplemental Fig. 3a). In line with the proliferative effect, p16INK4A and p14ARF, as markers of cell cycle arrest, were significantly decreased in cocktail-treated senescent cells compared to untreated (Fig. 2c and d). The expression of p14ARF was even depressed to a level comparable to that observed in non-senescent cells. Both markers were still less expressed after long-term cultivation (Supplemental Fig. 2b). Furthermore, qPCR-based analysis revealed that the cocktail was associated with a relative stabilization in telomere length compared to untreated cells (Fig. 2e). Notably, telomere length did not differ between the non-senescent and senescent cells following the treatment.

### Inflammation and DNA damage is improved by the pharmacological reprogramming approach *in vitro*

**Fig. 3:**
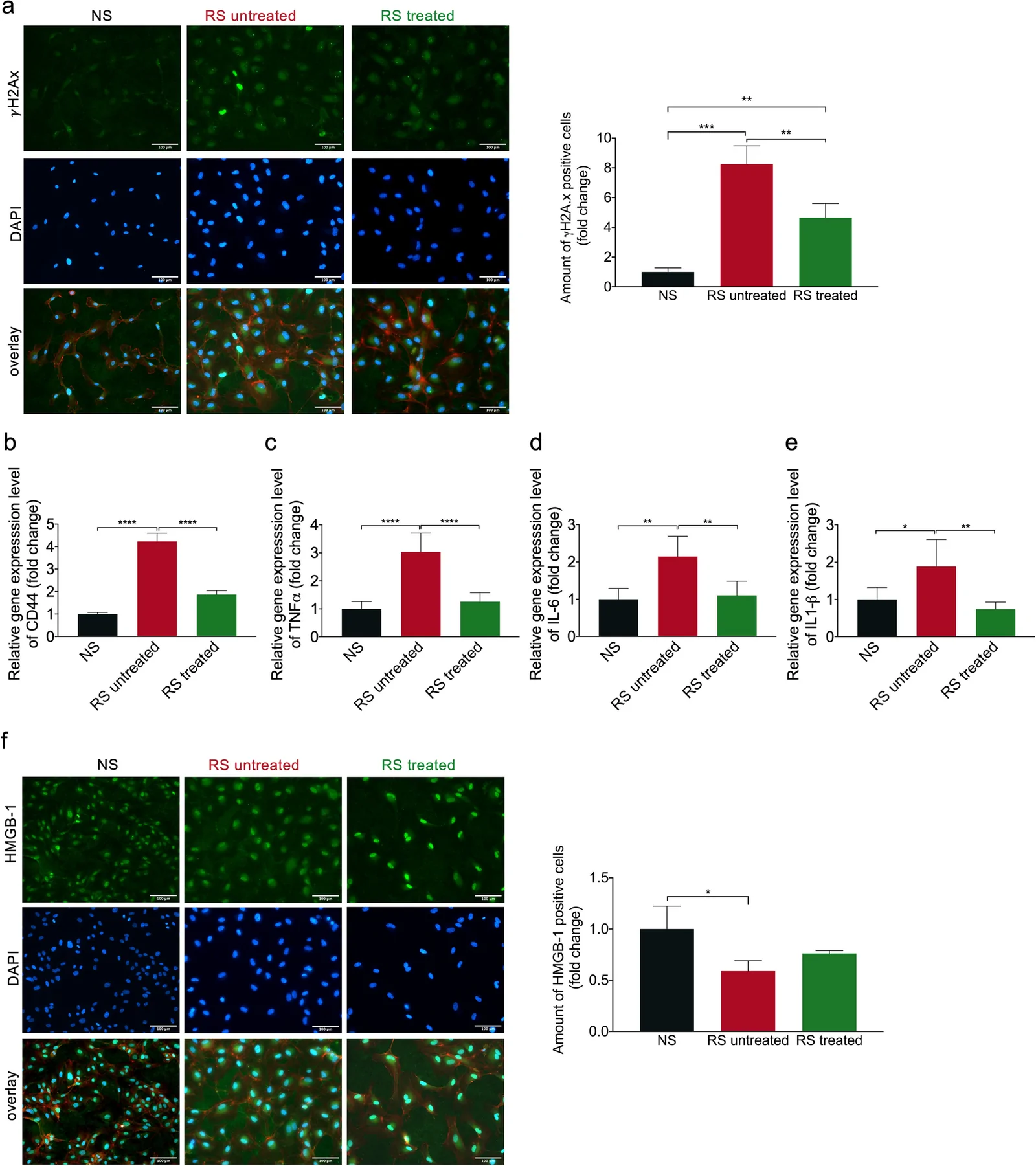
The pharmacological reprogramming cocktail attenuates features of the senescence-associated secretory phenotype in replicative senescent cells. **a** Quantification of yH2Ax-foci (green) to detect DNA double-strand breaks by immunofluorescence staining. The amount of yH2Ax foci is significantly reduced by the treatment (scale bar 100 µm; *n* = 3 biological replicates; one-way ANOVA). **b–e** Quantification of SASP components CD44, TNF-α, IL-6, and IL-1β reveals reduced relative gene expression levels compared to the untreated cells. There was no significant difference in gene expression between non-senescent (NS) and replicative senescent-treated (RS treated) endothelial cells (*n* = 3 biological replicates; one-way ANOVA). **f** Determination of HMGB-1 (green) release by immunofluorescence staining reveals no significant restoration of HMGB-1 levels after treatment (scale bar 100 µm; *n* = 3 biological replicates; one-way ANOVA). Statistical significance is indicated in the figures (*p < 0.05, **p < 0.01, ***p < 0.001, ****p < 0.0001)

The activation of the DNA damage response and the occurrence of DNA double-strand breaks are often described for senescent cells. DNA damage is decreased in the senescent cocktail-treated cells compared to the untreated senescent cells, as assessed by staining for γH2A.x, a histone that is phosphorylated upon DNA double-strand breaks to initiate various processes of the DNA repair system. This indicates that DNA repair mechanisms are initiated in senescent ECs by the cocktail (Fig. 3a). The release of cytokines and growth factors as part of an inflammatory process plays an important role in cellular senescence, a phenomenon called as the senescence-associated secretory phenotype (SASP). Components of the SASP measured here included the markers CD44, TNF-α, IL-6, and IL1-β. For all four markers, mRNA expression levels were reduced in the treated cells, with no elevation observed between the treated senescent cells and the non-senescent cells (Fig. 3b–e). A molecular regulator of these processes is HMGB-1. It is released from the cytosol of replicative senescent cells, binds to extracellular receptors, and initiates, among others, the regulation of cytokines such as IL-6. The release of HMGB-1 was successfully detected; however, it was not fully restorable in senescent-treated cells (Fig. 3f). One possible explanation might be that the timing after the cocktail application was not sufficient to yet restore HMGB-1 levels.

### The pharmacological reprogramming improves essential functional properties of senescent endothelial cells *in vitro*

**Fig. 4:**
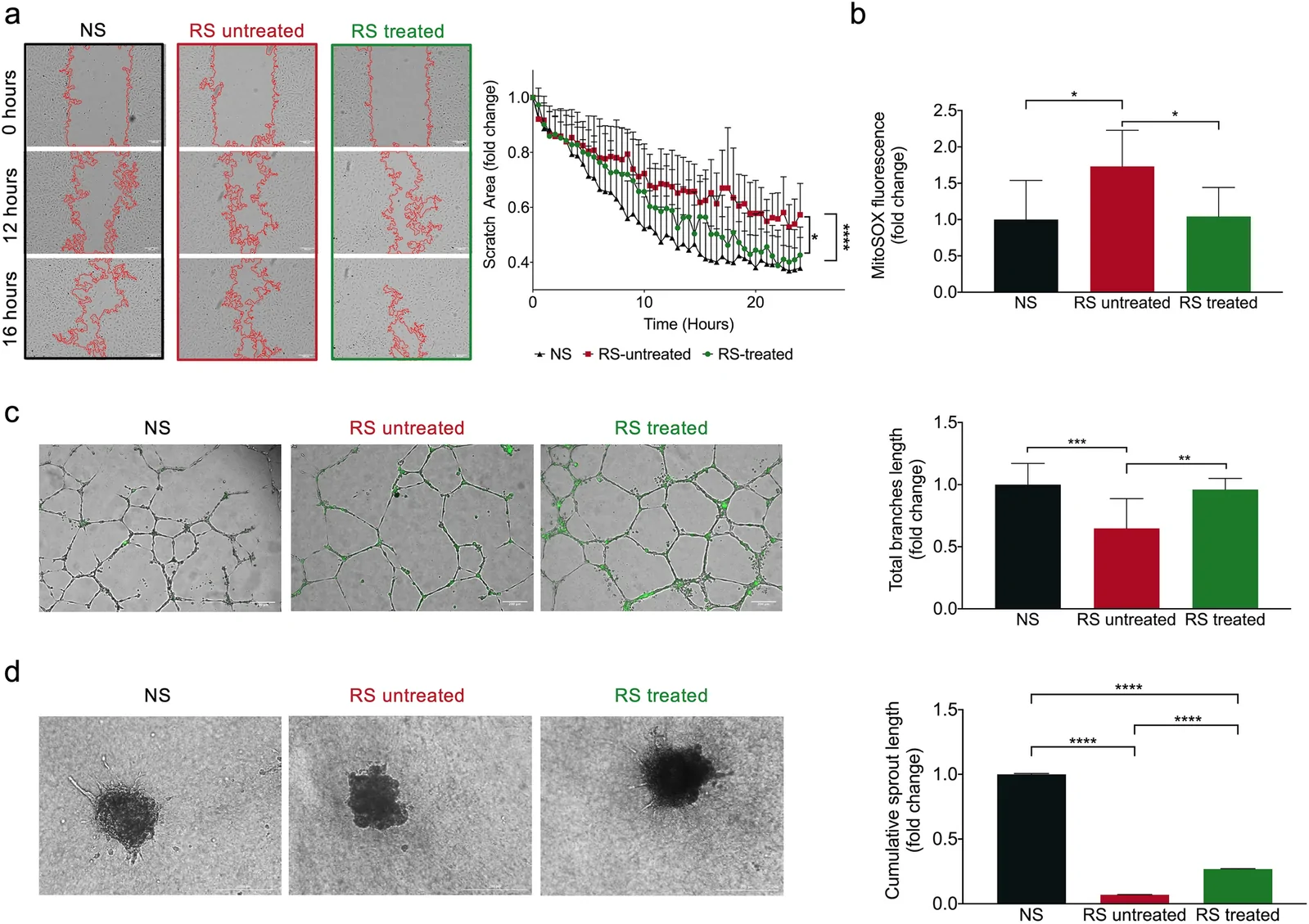
The angiogenic capacity of replicative senescent endothelial cells can be restored by a combinatorial treatment with VPA, Li2CO3 and Galunisertib. **a** Quantification of migration capacity by a scratch-wound assay. Wound closure is significantly faster in the replicative senescent-treated cells (scale bar 200 µm; *n* = 3 biological replicates; one-way ANOVA). **b** Mitochondrial superoxide is significantly reduced by the treatment measured by MitoSOX staining (*n* = 3 biological replicates; one-way ANOVA). **c** Performance of tube formation assay shows significantly longer total branch length in the treated cells that have a length comparable to those in the non-senescent cells (Scale bar 200 µm; *n* = 3 biological replicates; one-way ANOVA). **d** Cumulative sprout length in an endothelial cell spheroid sprouting assay showed significantly longer spheres in the treated cells (Scale bar 200 µm; *n* = 3 biological replicates; One-way ANOVA). Statistical significance is indicated in the figures (*p < 0.05, **p < 0.01, ***p < 0.001, ****p < 0.0001)

In addition to standard pathways that are often dysregulated in senescent cells, important functional properties of endothelial cells were analyzed *in vitro.* First, endothelial cell migration was measured by a scratch-wound assay (Fig. 4a). Wound closure occurred faster in the treated than in the untreated senescent cells. Also, migration capacity was again assessed after 28 days, still showing the improved migration capacity of the treated cells (Supplemental Fig. 3c). To determine the oxidative stress level, cells were stained for superoxide produced by mitochondria. The mitochondrial superoxide levels in the treated senescent cells were similar to those of the non-senescent cells and elevated in the senescent untreated cells (Fig. 4b). To study the angiogenic behavior of the cells, the ability of the cells to form sprouts and tubular networks was tested. Both assays showed an improved angiogenic behavior in the senescent cells receiving the pharmacological reprogramming cocktail (Fig. 4c and d).

**Fig. 5:**
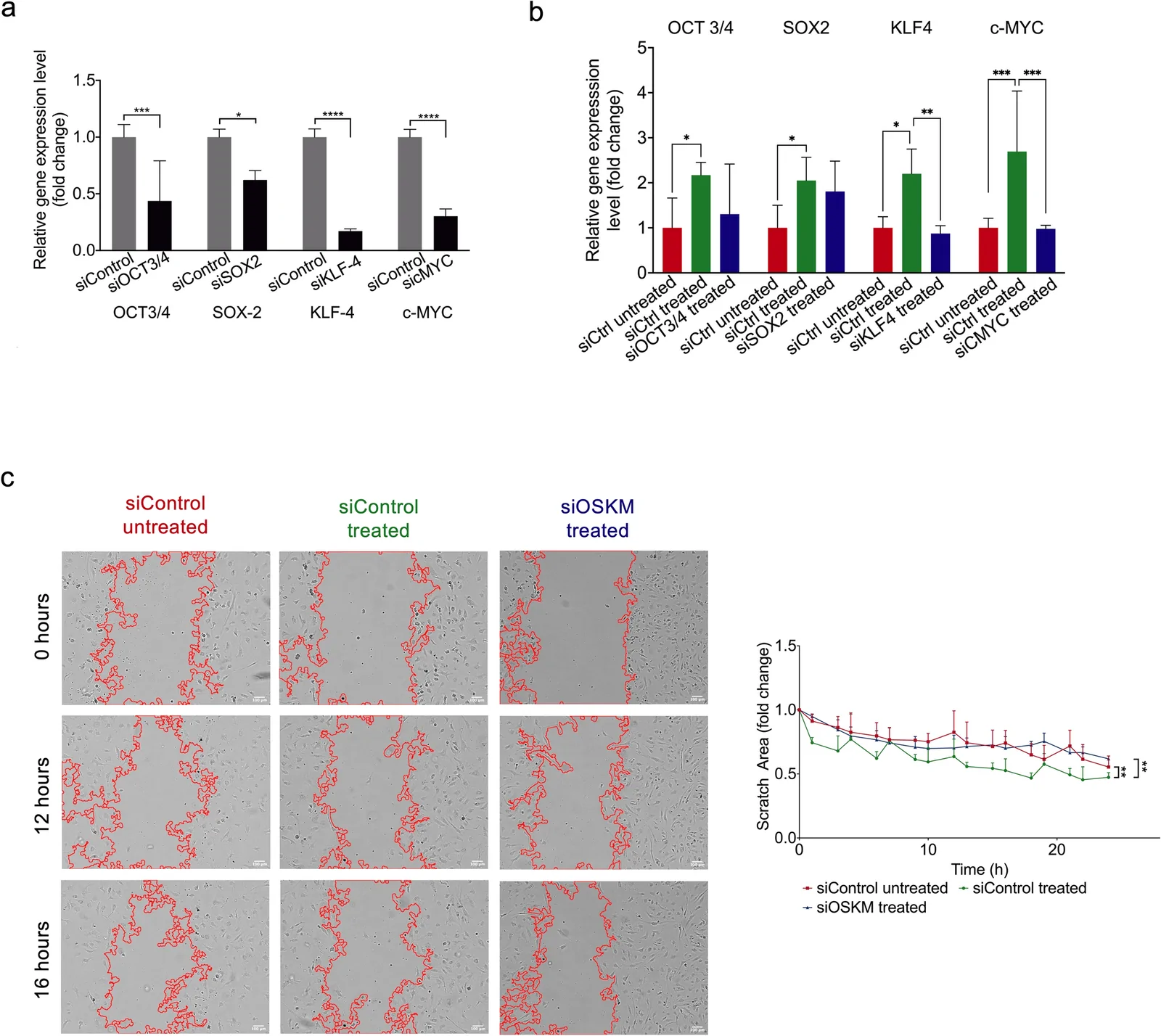
OSKM gene silencing by siRNA transfection prior to pharmacological reprogramming prevents positive effects of the treatment on migration. **a** Efficiency of gene silencing 24 h after siRNA transfection. All four genes are efficiently silenced by siRNA transfection (*n* = 3 biological replicates. Student’s *t *test). **b** Quantification of the relative gene expression level of OCT3/4, SOX2, KLF-4, and c-MYC after respective siRNA transfection and the 72-h treatment with VPA, Li2CO3, and Galunisertib (*n* = 5 biological replicates. one-way ANOVA with Dunnett’s T3 multiple comparison test). **c** Quantification of migration capacity after siRNA transfection and pharmacological reprogramming. Migration capacity of OSKM-silenced cells treated with the pharmacological cocktail (siOSKM treated) is at a comparable level as in the untreated control cells (siControl untreated) and differs significantly from the treated control cells (siControl treated) (scale bar 100 µm; *n* = 3 biological replicates; one-way ANOVA). Statistical significance is indicated in the figures (*p < 0.05, **p < 0.01, ***p < 0.001, ****p < 0.0001)

To further verify that attenuation of the senescent phenotype is directly dependent on OSKM overexpression and requires all four factors, OSKM were silenced by siRNA. Downregulation of OSKM by siRNA transfection was highly efficient (Fig. 5a). The parallel siRNA-mediated knockdown with the pharmacological treatment did restore OSKM levels to a comparable to that in the control cells (Fig. 5b), thereby indicating a counteracting effect by the treatment and the siRNAs regarding OSKM expression. Analysis of cellular functions by migration capacity showed no improvement in OSKM-silenced treated cells (Fig. 5c).

### The pharmacological reprogramming approach does not impair non-senescent cells

**Fig. 6:**
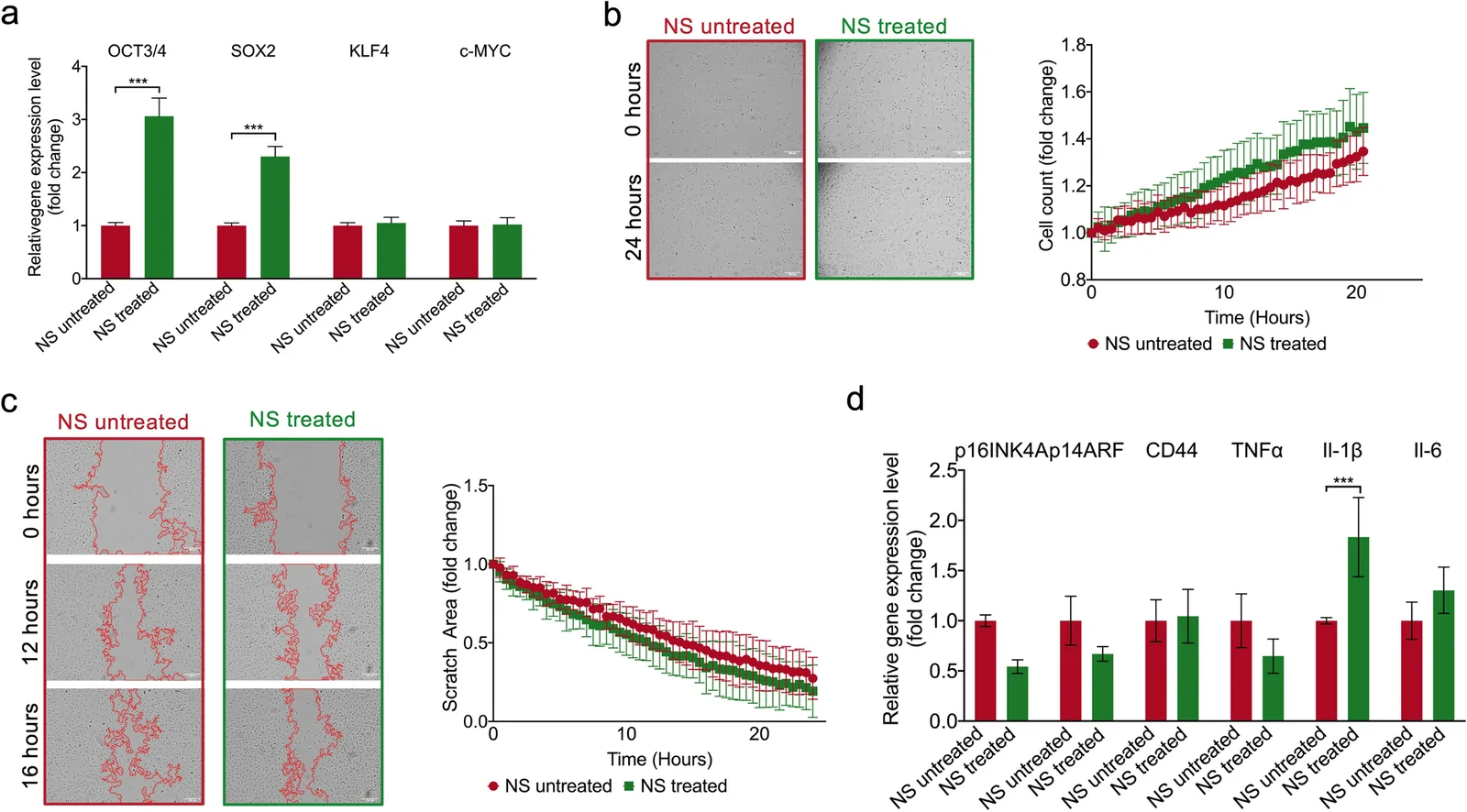
Non-senescent endothelial cells are not impaired by the pharmacological reprogramming approach. **a** The pharmacological reprogramming cocktail leads to an overexpression of OCT3/4 and SOX2 after a 72-h treatment (*n* = 3 biological replicates; Student’s *t *test). **b** Proliferation of non-senescent endothelial cells is not impaired by the pharmacological treatment (scale bar 200 µm; *n* = 3 biological replicates; two-way ANOVA). **c** The pharmacological treatment has no influence on the migration capacity (Scale bar 200 µm; *n* = 3 biological replicates; two-way ANOVA). **d** Treatment with the pharmacological reprogramming cocktail does not have an effect on common senescence markers, but induces a significant regulation of IL1-β (*n* = 3 biological replicates; Student’s *t *test). Statistical significance is indicated in the figures (*p < 0.05, **p < 0.01, ***p < 0.001, ****p < 0.0001)

In general, senescent ECs that accumulate in the vasculature are neighboring non-senescent ECs. An important criterion for an intervention targeting senescent cells is that the intervention does not harm or negatively impact non-senescent cells. To test this, non-senescent cells were treated with the same pharmacological cocktail according to the same protocol. Observations indicate that only two OSKM factors are induced by the cocktail in the non-senescent setting, namely OCT3/4 and SOX2 (Fig. 6a). KLF-4 and c-MYC show no regulation at all. To test for a functional impairment, a proliferation assay by live-cell count and a scratch-wound assay were performed (Fig. 6b and c). None of the assays showed any differences between the groups. Also, the cell cycle regulators p16INK4A and p14ARF, as well as the SASP components CD44, TNF-α, IL-6, and IL1-β were screened for their expression (Fig. 6d). No regulation of these factors was observable; only IL1-β expression was slightly increased in the treated cells.

To assess potential effects of the reprogramming cocktail on non-endothelial vascular cell types, complementary *in vitro* experiments were performed in replicative senescent vascular smooth muscle cells, a key neighboring cell population interacting closely with endothelial cells during angiogenesis and vascular remodeling. In contrast to endothelial cells, smooth muscle cells did not exhibit comparable molecular or functional responses to the pharmacological cocktail (Supplemental Fig. 4).

### Pharmacological reprogramming in a mice hind-limb ischemia model enhances angiogenic capacity *in vivo*

**Fig. 7:**
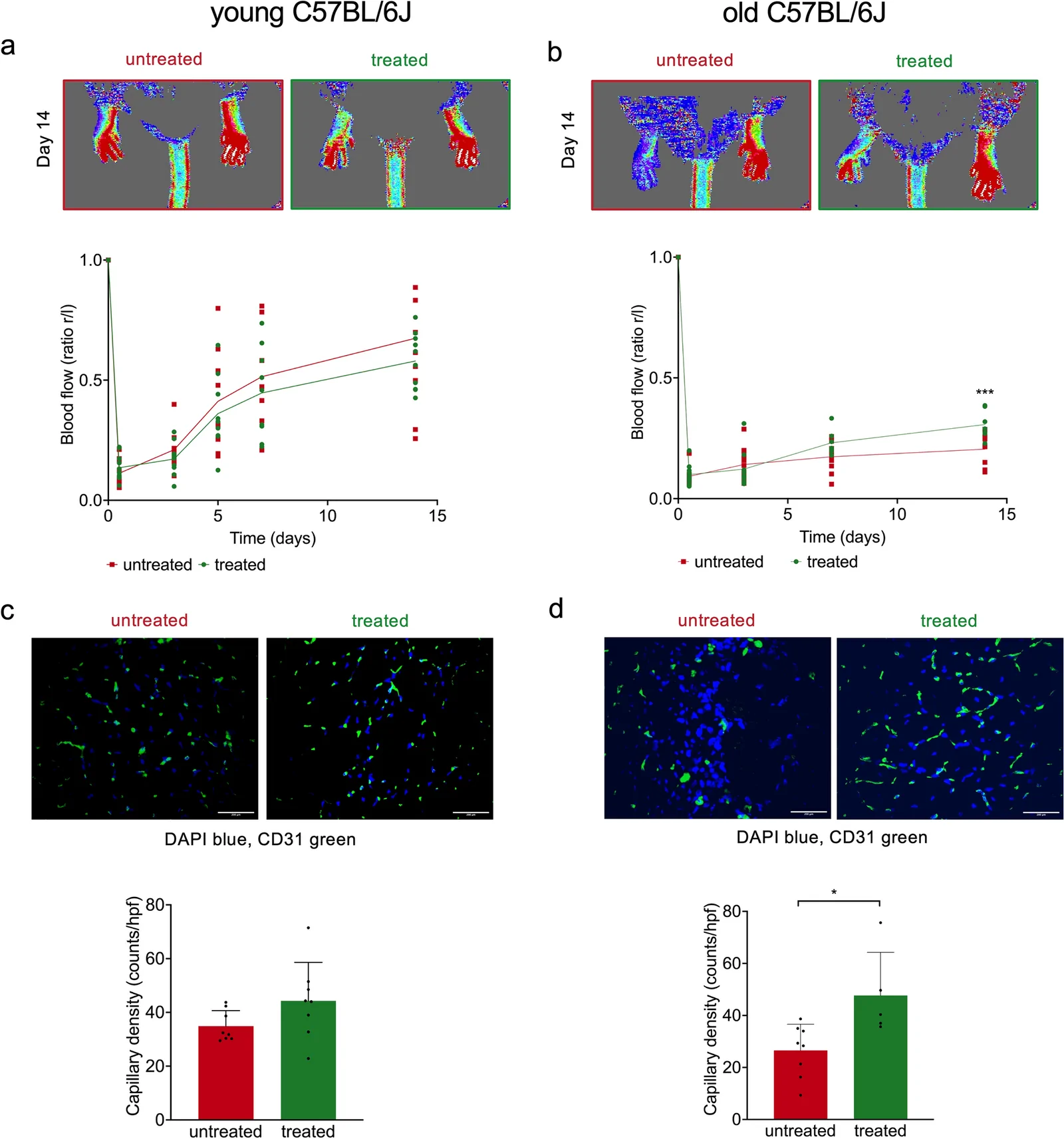
Treatment with the pharmacological reprogramming cocktail can improve angiogenesis *in vivo* after hind-limb ischemia. **a** In young C57BL/6 J animals (3 months), the cocktail does not affect blood flow (two-way ANOVA). **b** Blood-flow ratio is significantly improved in old C57BL/6 animals (21 months) receiving a treatment with the pharmacological reprogramming cocktail (two-way ANOVA). **c** Capillary density (green IF staining by CD31) in the gastrocnemius muscle is comparable in untreated and treated young C57BL/6 J animals on day 14 after surgical intervention (scale bar 200 µm; Student’s *t *test). **d** Capillary density (CD31 staining green) is significantly enhanced in old C57BL/6 animals by the pharmacological treatment (Scale bar 200 µm; Student’s *t *test). Statistical significance is indicated in the figure (*p < 0.05, **p < 0.01, ***p < 0.001, ****p < 0.0001)

The hind-limb ischemia model is a well-established mice model to study vascular regeneration. By permanently ligating the femoral artery distal to the origin of the deep branch, the model offers the opportunity to study arteriogenesis in the femoral collaterals as well as angiogenesis in the distal muscle. The ligation leads to ischemia in the distal muscle, the gastrocnemius, and thereby triggers the angiogenic response. The experiments were conducted in young (12 weeks) and old animals (21 months), and the pharmacological cocktail was administered i.p. directly after surgical intervention, on day 1, and 3. Laser-Doppler imaging revealed that the cocktail did not affect blood flow in the young animals. However, blood flow was significantly improved in the old animals receiving the cocktail treatment (Fig. 7a and b). Improved blood flow in aged animals receiving the treatment was supported by increased capillary density, measured by staining for CD31 (Fig. 7c and d). Neither the analysis of blood parameters nor the measurement of weight or the quantification of the necrotic score of the ligated foot did reveal any differences between the control and treatment groups (Supplemental Fig. 5a to c). Additional analysis studied OSKM expression in the endothelial cells (CD146^+^ cells) on day 4 after surgical intervention. Whereas Oct3/4 and SOX2 were still upregulated in the treated animals, KLF-4 and cMYC expression dropped to a physiological level, thereby showing the same effect as *in vitro* (Supplemental Fig. 6 A and B). In contrast, no corresponding regulation was detected in the non-endothelial cell fraction (CD146^−^ cells).

## Discussion

Cardiovascular diseases remain the leading cause of death worldwide despite substantial progress in CVD treatment. CVDs are characterized by endothelial dysfunction, partly due to the presence of senescent endothelial cells. Among other aspects, these cells further limit the regenerative potential and angiogenic capacity of the vasculature. To overcome this hurdle, we here established a pharmacological, partial reprogramming strategy based on the timely restricted overexpression of the Yamanaka-factor OSKM to rejuvenate the senescent EC and improve tissue function and vascularization after vascular damage.

We could demonstrate that OSKM overexpression in response to cocktail application was controllable in a time-dependent manner leading to functional improvements of senescent-treated EC on a short- and long-term basis compared to untreated EC *in vitro*. Partly, this effect even exceeded the function of comparable non-senescent endothelial cells. Additionally, we proved a positive effect on blood flow and capillary density *in vivo* after hind-limb ischemia.

An aged organism is characterized by the loss of the regenerative and angiogenic potential [9, 22, 49, 58]. In contrast, animals preserving their regenerative potential do not show any signs of aging [59]. The so-called reprogramming genes, among others, OCT3/4, c-MYC, KLF-4, and SOX2, play a major role in cellular regeneration [6, 55], helping cells to enter a reprogramming-like state after tissue damage [26]. We show a lack of initiation of regeneration due to cellular damage in replicative senescent endothelial cells by exploiting a well-established CVD-relevant damage model, namely oxidative stress damage (modeling cellular stress and influencing vascular reactivity) [11].

We then showed that by initiating physiological reprogramming as observable in non-senescent cells with a short artificial stimulus, the senescent cells will also start a partial reprogramming process to regain cellular function. So far, most methods working with cellular reprogramming, achieve an overexpression of OSKM by viral transfection to generate iPSC leading to a shift in cellular fate and potentially promoting genomic instability and teratoma formation [35, 53, 62]. Whereas a virally induced long-term induction of OSKM *in vivo* led to teratoma formation and altered DNA methylation patterns [1, 44], a cyclic induction of OSKM can increase lifespan and improve regeneration and signs of aging while reducing the risk of teratoma formation [42]. Based on these considerations, we decided to use small pharmacological molecules that are easier to handle in application and concentration to induce partial reprogramming without entry into pluripotency, thereby preserving endothelial identity and function. Although the compounds used in this study are individually FDA-approved drugs in their respective concentrations, their combined use, dosing regimen, and therapeutic context are not approved and therefore do not imply direct clinical applicability.

The pharmacological cocktail is composed of three compounds: valproic acid, lithium carbonate, and Galunisertib. All three compounds are not only known to support cellular reprogramming [54, 57, 65] but are also shown to have a potential role in cellular aging processes by interacting with aging-related pathways [23, 51, 56]. Valproic acid, as a histone deacetylase inhibitor, supports the development of neural stem and progenitor cells [15, 61]. Lithium carbonate enhances iPSC generation [7, 43, 57] and blocks the formation of a GSK-3/p53 complex inhibiting senescence accumulation [51, 66]. Galunisertib, as a transforming growth factor-β (TGF-β) inhibitor, can sustain pluripotency [64], whereas the activation of TGF-β blocks reprogramming [34]. A combination of all three compounds was shown to promote cellular reprogramming and improve liver regeneration and hepatic function *in vivo* [55]*.*

In our study, we demonstrated that efficient overexpression of OSKM by a combination of small pharmacological compounds is possible and that this regulation occurs in a time- and concentration-dependent manner, thereby reducing the risk of teratoma formation and genomic instability, as observed with viral overexpression. These risks were further reduced as the reprogramming process was only partial as the cells retained their endothelial cell identity. However, this short overexpression was sufficient to improve observed cellular functions as well as gene expression of senescence markers. By OSKM silencing, parallel to pharmacological reprogramming, the importance of all four factors for sufficient reprogramming was proven. Here, we could show that the expression of cell cycle genes p16INK4A and p14ARF can be reduced by the pharmacological cocktail. Comparable results were made in iPSC research when reprogramming senescent fibroblasts [29, 37] and in an *in vitro* model enabling partial reprogramming by doxycycline-inducible OSKM expression [42]. DNA damage-associated markers were attenuated by the pharmacological treatment in our senescent ECs, in contrast to iPSC-based reprogramming approaches, where accumulation of DNA damage and mutations in mitochondrial DNA has been reported to persist [35]. Telomere length estimates assessed by qPCR were stabilized in response to the pharmacological cocktail. Similar changes have been reported in the context of iPSC generation, where telomere maintenance or elongation can occur [29, 37]. However, as qPCR-based approaches provide relative rather than absolute measurement of telomere length, these findings should be interpreted with appropriate caution. Whether these changes involve altered telomerase activity or other telomere maintenance mechanisms will require further investigation. The SASP is an important feature for successful cellular reprogramming [40, 48]. Efficient attenuation of several SASP components was observed, consistent with previous reprogramming-based approaches for IL-6 [42]. In contrast, HMGB-1 localization was not restored by the pharmacological cocktail, indicating that not all senescent-associated features were completely normalized. An explanation might be that the time between the treatment and the assessment of HMGB-1 levels was not sufficient to restore intracellular HMGB-1 levels. Mitochondrial function was partially improved by the partial reprogramming, comparable to reports from iPSC-based reprogramming where enhanced mitochondrial function has been described [37, 52]. Although mitochondrial superoxide was assessed using a qualitative fluorescence-based approach, the modulation of endothelial mitochondrial ROS has been shown to critically influence endothelial resilience and vascular function under ischemic stress [5, 30].

In additional experiments, angiogenic and regenerative capacity were shown to be restored *in vivo*. In the murine hind-limb ischemia model, angiogenesis and early arteriogenic remodeling predominantly occur within the first 2 weeks after ischemia induction, whereas later time points mainly reflect vessel maturation and stabilization rather than dynamic endothelial plasticity. Consistent with this, longitudinal perfusion studies indicate that perfusion recovery increases primarily during the early post-ischemic phase and subsequently approaches a plateau [5, 30]. Improved blood flow and capillary density in old mice receiving a treatment with the pharmacological cocktail encourage observed *in vitro* results. However, endothelial cells constitute a functionally heterogeneous population, which may contribute to the partial and marker-specific nature of the observed OSKM response[51]. Comparable results have been shown for the application of the cocktail after liver injury regardless of age [55] and after partial induction of OSKM expression by doxycycline administration in LAKI 4F mice, where attenuation of aging-associated hallmarks was demonstrated by β-galactosidase staining [42]. Furthermore, our findings are consistent with previous work, which demonstrates that partial reprogramming strategies can enhance endothelial angiogenic capacity. In particular, Yin et al. demonstrated that partial OSKM-mediated reprogramming of endothelial cells promotes angiogenesis in the coronary circulation, enabling the generation of induced vascular progenitor cells with enhanced vascular plasticity [63]. While this study focused on a distinct vascular bed and employed a cell-based reprogramming strategy aimed at coronary collateral growth, it supports the broader concept that partial reprogramming can modulate endothelial function and angiogenic responses. Our data extend these observations to age-associated endothelial dysfunction and impaired angiogenesis in peripheral ischemia, demonstrating that transient, pharmacological induction of partial reprogramming is sufficient to improve angiogenic outcomes *in vivo*.

In the present study, we observed sustained functional improvements *in vitro* following a single, transient exposure to the pharmacological cocktail, without evidence of adverse effects during extended culture. Notably, the treatment did not affect non-senescent endothelial cells, suggesting a degree of context dependency and arguing against generalized activation or uncontrolled cellular responses. Given the limited availability of data on partial reprogramming approaches aimed at rejuvenating senescent endothelial cells, our findings are primarily discussed in comparison to iPSC-based reprogramming strategies derived from (senescent) cells. Beyond cell-autonomous effects, repeated or systemic exposure to reprogramming-inducing compounds raises additional safety considerations. Partial reprogramming approaches have been associated with potential risks such as aberrant cell cycle re-entry, epigenetic instability, or unintended effects on non-target cell populations, depending on dose, duration, and tissue context. In the present study, systemic exposure was transient, and no overt adverse effects were detected *in vivo*. Although the reprogramming cocktail was administered systemically, the preferential response observed in endothelial cells *in vivo* and the lack of comparable effects in smooth muscle cells *in vitro* support a primary endothelial contribution to the angiogenic effects observed. Nevertheless, improved endothelial function may secondarily modulate post-ischemic tissue remodeling through paracrine interactions with inflammatory, stromal, or regenerative cell populations. Such indirect effects are not mutually exclusive with a primary endothelial mechanism and may contribute to the overall improvement in blood flow recovery and angiogenesis. A comprehensive assessment of potential cumulative or long-term consequences following repeated or prolonged systemic administration will require dedicated *in vivo* studies with extended follow-up and remains an important focus for future investigation. Especially as endothelial function may secondarily influence surrounding vascular cells through paracrine crosstalk, consistent with emerging evidence highlighting functional interactions between endothelial cells and pericytes in the regulation of vascular stability and perfusion [18]

By our research, we could demonstrate that endothelial cell senescence and cellular reprogramming can be linked. Thereby, a short overexpression of OSKM by pharmacological compounds is already sufficient to partially restore endothelial cell function *in vitro* and *in vivo* and to attenuate multiple senescence-associated cellular features. The partial and timely restricted overexpression of OSKM supports a partial reprogramming strategy where the cells sustain their endothelial cell identity which further reduces the risk of teratoma formation and genomic instability. Altogether, targeting senescent endothelial cells by a partial reprogramming strategy with small molecules might present a promising strategy to treat endothelial dysfunction in CVD. While the use of compounds individually approved for other indications highlights a theoretical translational potential, further preclinical and clinical studies will be required to assess safety, efficacy, and regulatory feasibility before clinical application can be considered.

## Supplementary Information

Below is the link to the electronic supplementary material.

Supplementary file1 (DOCX 98819 KB)

## Data Availability

The datasets generated and/or analyzed during the current study are available from the corresponding author on reasonable request.
