# Supplementary material for: Functional rejuvenation of endothelial cell aging by transient reprogramming
Source: Basic Res Cardiol. 2026 Jun 26;121(5):1029–45. doi: 10.1007/s00395-026-01192-7 (PMC13593674; doi:10.1007/s00395-026-01192-7)
Supplement: Supplementary file 1 [file 395_2026_1192_MOESM1_ESM.docx]

# Supplementary Material

# Supplementary Table S1: Used siRNA for transfection experiments

| Target Gene | siRNA name | Sequence/ Specification | Manufacturer |
| --- | --- | --- | --- |
| c-MYC | ON-TARGET plus Human MAC (4609) siRNA – SMARTpool | L-003282-02-0005 | Dharmacon, Horizon Discovery, Lafayette, US |
| KLF4 | ON-TARGET plus Human KLF4 (9314) siRNA - SMARTpool | L-005089-00-0005 | Dharmacon, Horizon Discovery, Lafayette, US |
| POU5F1 (OCT3/4) | Hs_POU5F1_2 Flexitube siRNA | SI00690382 | Qiagen, Hilden, Germany |
| SOX2 | Hs_SOX2_5 Flexitube siRNA | SI04136167 | Qiagen, Hilden, Germany |
| Scrambled control | ON-TARGET plus Non-targeting Pool | D-001810-10-05 | Dharmacon, Horizon Discovery, Lafayette, US |
| Scrambled control | Negative control siRNA | 1022076 | Qiagen, Hilden, Germany |

# Supplementary Table S2: Used primers for qRT-PCR

| Gene |  | Sequence |
| --- | --- | --- |
| OCT3/4 | for | AGC GAA CCA GTA TCG AGA AC |
|  | rev | TTA CAG AAC CAC ACT CGG AC |
| SOX2 | for | GGG AAA TGG GAG GGG TGC AAA AGA GG |
|  | rev | TTG CGT GAG TGT GGA TGG GAT TGG TG |
| CD44 | for | TGGCACCCGCTATGTCGAG |
|  | rev | GTAGCAGGGATTCTGTCTG |
| TNFα | for | GGCGTGGAGCTGAGAGATA |
|  | rev | CAGCCTTGGCCCTTGAAGA |
| IL1β | for | AAAGCTTGGTGATGTCTGGTC |
|  | rev | GGACATGGAGAACACCACTTG |
| KLF4 | for | TCT CAA GGC ACA CCT GCG AA |
|  | rev | TAG TGC CTG GTC AGT TCA TC |
| GAPDH | for | TGCACCACCAACTGCTTAGC |
|  | rev | GGCATGGACTGTGGTCATGAG |
| FBX07 | for | GCTCGCACCTGAGGCAGTCC |
|  | rev | GTCTCTTCATCTCCAGTGAGGGG |
| c-MYC | for | GCG TCC TGG GAA GGG AGA TCC GGA GC |
|  | rev | TTG AGG GGC ATC GTC GCG GGA GGC TG |
| p16INK4A | for | GGG GGC ACC AGA GGC AGT |
|  | rev | GGT TGT GGC GGG GGC AGT T |
| p14ARF | for | CCC TCG TGC TGA TGC TAC TG |
|  | rev | CAT CAT GAC CTG GTC TTC TAG GAA |
| ActB | for | CCT CGC CTT TGC CGA TCC G |
|  | rev | CGA CGA GCG CGG CGA TAT CAT |

# Supplementary Table S3: Used antibodies for Western blot analysis (WB) and immunofluorescence staining (IF)

| Antibody | Company | Order number | Application | Dilution |
| --- | --- | --- | --- | --- |
| Phalloidin-TRITC | Sigma | P1951 | IF | 1:100 |
| CD31 | Cell Signaling | 3528S | IF | 1:100 |
| HMGB-1 | Santa Cruz | sc-135809 | IF | 1:1000 |
| γH2Ax | Cell Signaling | 9718 | IF | 1:200 |
| Oct3/4 | Cell Signaling | #2840 | WB | 1:1000 |
| c-Myc | Cell Signaling | #5605 | WB | 1:1000 |
| GAPDH | Santa Cruz | sc-32233 | WB | 1:5000 |
| KLF4 | abcam | ab72543 | WB | 1:1000 |
| SOX2 | Cell Signaling | #4900 | WB | 1:1000 |

# Supplementary Figure 1: To validate mitochondrial superoxide specificity, cells were treated with Rotenone or MitoTEMPO as positive and negative controls for MitoSOX fluorescence. Non-senescent cells were treated with rotenone to induce mitochondrial superoxide and a positive MitoSOX signal, whereas replicative senescent cells were treated with MitoTEMPO to counteract the positive signal.

# Supplementary Figure 2: Pharmacological treatment does not influence cell morphology or endothelial cell identity. (A) Brightfield images of non-senescent (NS), replicative senescent untreated (RS untr), and replicative senescent treated (RS treated) cells. (B) CD31-staining of non-senescent (NS), replicative senescent-untreated (RS untreated), and replicative senescent-treated (RS treated) for DAPI (blue), CD31 (green) and Phalloidin (red). (B) Quantification of nuclear size by measurement of DAPI staining. (D) Quantification of CD31-positive stained cells to DAPI-staining to assess EC identity.

# Supplementary Figure 3: Determination of the long-term in vitro effect 28 days after initial treatment with the pharmacological reprogramming cocktail. (A) Quantification of the gene expression of SASP and cell cycle genes that are associated with cellular senescence. The expression of p16INK4A and p14ARF is still significantly downregulated in the once treated senescent cells (RS-trea) compared to the untreated senescent cells (RS-untr). n=3 p16INK4A **p=0.0093, p14ARF *p=0.0102 (B) Determination of proliferation by cell count via life cell imaging over 24 hours. The once treated senescent cells (RS-trea) do still proliferate better than the untreated senescent cells (RS-untr). n=3 ****p<0.0001 (C) The scratch-wound assay demonstrates that senescent treated cells (RS-trea) have a better migration capacity than senescent untreated cells (RS-untr) even 28 days after initial treatment. n=3 ****p<0.0001

# Supplementary Figure 4: The pharmacological treatment does not influence replicative senescent smooth muscle cells on (A) mRNA expression levels of OSKM 72 hours after initial treatment. B) mRNA expression of senescence-associated genes and C)-E) the functional properties of the replicative senescent smooth muscle cells were not affected by the treatment.

# Supplementary Figure 5: Analysis of health parameters reveals no influence by the treatment. (A) Determination of blood parameters from young (3 months) and old (21 months) animals receiving the pharmacological reprogramming cocktail after hind-limb ischemia. All parameters are represented as median (IQR). (B) Measurement of weigth (g) of each mice. (C) Determination of nectrotic score of each operated animal on the ligated foot.

# Supplementary Figure 6: The pharmacological treatment with VPA, Li2CO3 and Galunisertib leads to an application-dependent overexpression of Sox-2 and OCT3/4 in a hind-limb ischemia model in A) CD146^+^-cells in 21-months old C57BL/6J mice on day 4 after hind-limb ischemia surgery. For KLF4 and cMYC no significant regulation was observable. In B) CD146^—^cells, no such regulation was observable. n=5-6 *p<0.05
